# ^18^F‐FDG PET/CT imaging: A supplementary understanding of pulmonary sclerosing pneumocytoma

**DOI:** 10.1111/1759-7714.13100

**Published:** 2019-05-27

**Authors:** Jie Xu, Youwen Dong, Guotao Yin, Wei Jiang, Zhen Yang, Wengui Xu, Lei Zhu

**Affiliations:** ^1^ Department of Senior Ward, National Clinical Research Center for Cancer, Tianjin Key Laboratory of Cancer Prevention and Therapy, Tianjin's Clinical Research Center for Cancer Tianjin Medical University Cancer Institute and Hospital Tianjin China; ^2^ Imaging Center The Affiliated Hospital of Jining Medical University Jining China; ^3^ Department of Molecular Imaging and Nuclear Medicine, National Clinical Research Center for Cancer, Tianjin Key Laboratory of Cancer Prevention and Therapy, Tianjin's Clinical Research Center for Cancer Tianjin Medical University Cancer Institute and Hospital Tianjin China

**Keywords:** ^18^Fluorine 2‐fluoro‐2‐deoxy‐d‐glucose, positron emission tomography and computed tomography, pulmonary sclerosing pneumocytoma

## Abstract

**Background:**

We sought to investigate the clinical features and ^18^F‐FDG PET/CT characteristics of pulmonary sclerosing pneumocytoma (PSP).

**Methods:**

We retrospectively reviewed and comparatively analyzed ^18^F‐FDG PET/CT imaging results of 22 patients with diagnosed PSP in our hospital from November 2009 to September 2015.

**Results:**

The SUV_max_ in tumors was positively correlated with tumor size in typical PSPs (R = 0.806, R^2^ = 0.650, *P* = 0.001); however, the SUV_max_ in tumors had no significant correlation with tumor size of atypical PSPs (R = 0.479, R^2^ = 0.229, *P* = 0.162), and the degree of correlation between them attenuated when atypical PSPs were included (R = 0.518, R^2^ = 0.268, *P* = 0.011). A majority (90%) of atypical PSPs were found in males. Symptomatic patients showed a higher SUV_max_ than the asymptomatic group (5.68 ± 3.63 vs. 2.76 ± 1.18, respectively, *P* = 0.002).

**Conclusion:**

Tumor size and clinical features may be associated with increased FDG uptake in PSPs. Morphological differences may affect the correlation between tumor size and SUV_max_ in PSPs. The atypical form of PSP may be more common in men.

## Introduction

Pulmonary sclerosing pneumocytoma (PSP), is a rare benign lung tumor formerly known as sclerosing hemangioma (Liebow and Hubbell^1^ due to its prominent sclerosis and vascularization. Chan *et al.* 2 first suggested that the name pneumocytoma was more appropriate for this tumor. The term sclerosing hemangioma was subsequently renamed sclerosing pneumocytoma in the 2015 World Health Organization Classification of Lung Tumors.^3^

Pathologically, PSP is typically composed of solid cellular areas, papillary structure, sclerotic regions, and dilated blood‐filled spaces. The four major structures may vary in their proportions, possibly resulting from the migration of hyperplasia from the these areas.^4^

Several studies have been conducted to investigate PSPs using ^18^F‐FDG PET/CT imaging. Results indicated that low‐to‐moderate uptake of PSPs were found by ^18^F‐FDG PET/CT imaging, and tumor size of PSPs was positively correlated with SUV_max_ in these tumors.^5^, ^6^, ^7^ Other studies also showed no correlation between tumor size and SUV_max_ in these tumors.^8^

The purpose of our study was to investigate the characteristics of PSPs using PET/CT scans in our hospital, in order to obtain comprehensive understanding of this rare benign neoplasm.

## Methods

### Patients

A total of 136 patients comprising 113 females (83.1%) and 23 males (16.9%) ranging in age from 15 to 72 years (median, 51 years) were diagnosed with PSPs in our hospital between November 2009 to September 2015, among which 22 cases underwent PET/CT scans. A total of 21 cases underwent surgical resection, and one case was diagnosed by fluoroscopy‐guided core‐needle biopsy, resulting in a total of 23 PSP tissue samples (one case had two lesions, in the right upper lobe and right middle lobe, respectively). A 68‐year‐old woman with PSP underwent two PET/CT scans on 21 January 2014 and 15 March 2015. Another patient underwent a dual‐time‐point ^18^FDG‐PET/CT scan, through which we acquired PET/CT images of the chest by delayed scanning 75 minutes after routine scanning. Detailed PET/CT findings in PSPs are listed in Table 1. A total of 14 of the tumors were found incidentally on chest radiographs; the other nine patients presented with symptoms, four with fever, four with chest (back) pain, seven with cough, and two with hemoptysis.

**Table 1:** 18F‐FDG PET/CT findings in PSP

	Age	Sex	Size (cm)	Qualitative assessment†	SUV_max_	Morphology**‡**
1	40	F	3.3	Intense uptake	3.7	1
2	64	M	2.5	Moderate uptake	2.4	2
3§	65	M	1 (1.4)	Moderate (intense) uptake	2.3 (3.2)	2
4	62	M	3.6	Intense uptake	6.7	2
5	51	M	6.5	Intense uptake	8.3	2
6	63	F	3.9	Intense uptake	4.7	1
7	70	M	3.5	Moderate uptake	1.6	2
8¶	17	F	4	Intense uptake	4 (4.8)	1
9	48	F	2.3	Moderate uptake	1.8	1
10	34	F	2.2	Intense uptake	4.4	1
11	59	F	1.0	No uptake	0.8	1
12	61	M	1.8	Intense uptake	8.9	2
13††	68	F	2.0 (2.2)	No (moderate) uptake	1.0 (2.2)	1
14	47	M	1.6	Intense uptake	2.9	2
15	46	M	0.9	Intense uptake	3.9	2
16	58	M	3.7	Intense uptake	12.5	2
17	70	F	0.6	No uptake	0.8	1
18	53	F	1.7	Intense uptake	2.7	1
19	47	F	4.0	Intense uptake	3.5	1
20	46	M	1.8	Intense uptake	2.6	1
21	58	F	1.6	Intense uptake	2.7	1
22	56	F	2.9	Intense uptake	3.1	1

† Intense uptake: SUV_max_ ≥ 2.5, moderate uptake: 2.5 > SUV_max_ ≥ 1.5, no uptake: SUV_max_ < 1.5.

‡ We defined the PSP that was smooth‐edged and round, or oval in shape as “1,” otherwise as “2.”

§ In Case 3, there are two PSP lesions in the right upper lobe and right middle lobe of the lung, of which the sizes and FDG uptake are described above, respectively.

¶ In Case 8, a delayed PET/CT scan (75 minutes after the first scan) was performed, resulting in a slightly increased SUV_max_ (in brackets).

†† Case 13 had two PET/CT scans on 21 January 2014 and 15 March 2015. Both the size and SUV_max_ of the PSP had increased by the second scan.

### PET/CT examination and image analysis

^18^F‐FDG‐PET/CT scanning was conducted using a Discovery ST PET/CT scanner (GE Healthcare, Milwaukee, WI, USA). Patients were required to fast for 6 hours before the scan. Serum glucose was monitored closely before the intravenous injection of ^18^F‐FDG. The administered activity of the radiotracer was 4.1–4.8 MBq (0.11–0.13 mCi) per kilogram of bodyweight. Scanning was conducted from the mid‐thigh to the vertex approximately one hour after ^18^F‐FDG injection. CT scanning was performed with the following parameters: current, 120–170 mA; voltage, 120 kV; slice thickness, 5 mm or 3.75 mm; and reconstruction interval, 5 mm or 3.75 mm. Attenuation‐corrected PET images were gathered at 2 minutes per frame, and were reconstructed with iterative algorithms. A non‐contrast CT scan targeted at the lung nodule with a slice thickness of 1.25 mm was also obtained.

The images were reviewed by three senior nuclear medicine radiologists on an Advantage Workstation (Version 4.4, GE Healthcare, Milwaukee, WI, USA). Suspicious cases were resolved by discussion. The following metabolic parameters were measured: the maximal standardized uptake value (SUV_max_) of the lung lesion. SUVs were automatically generated via software using the following equation: SUV = radioactivity concentration/ (injected activity/bodyweight).

### Statistical analysis

Statistical analysis was carried out using SPSS17.0 (Chicago, IL, USA). Data was presented as mean ± standard deviation. Pearson's correlation and linear regression analysis were used for analyzing the relation of the tumor size and SUV_max_. Student *t*‐test was used for comparing tumor size and SUV_max_ of the two groups. Ninety five percent A confidence level of 95% was selected to determine the significant difference between the two groups, and *P* < 0.05 showed a significant difference statistically.

## Results

### ^18^F‐FDG PET/CT findings

A total of 11 men and 11 women (aged: 53.77 ± 12.76 years, range 17–70 years) underwent PET/CT scans. Pathology of the 22 patients confirmed 23 PSP lesions. The maximal diameter of the 23 PSPs range from 0.6 to 6.5 cm (2.64 ± 1.31 cm). The SUV_max_ ranged from 0.8–12.5 (3.84 ± 2.82). A total of 69.5% of the PSPs (16/23) showed intense uptake (SUV_max_ > 2.5). Three PSPs (diameters of 1 cm, 0.6 cm and 2 cm, respectively) showed no uptake (SUV_max_ < 1.5).

**Figure 1:**
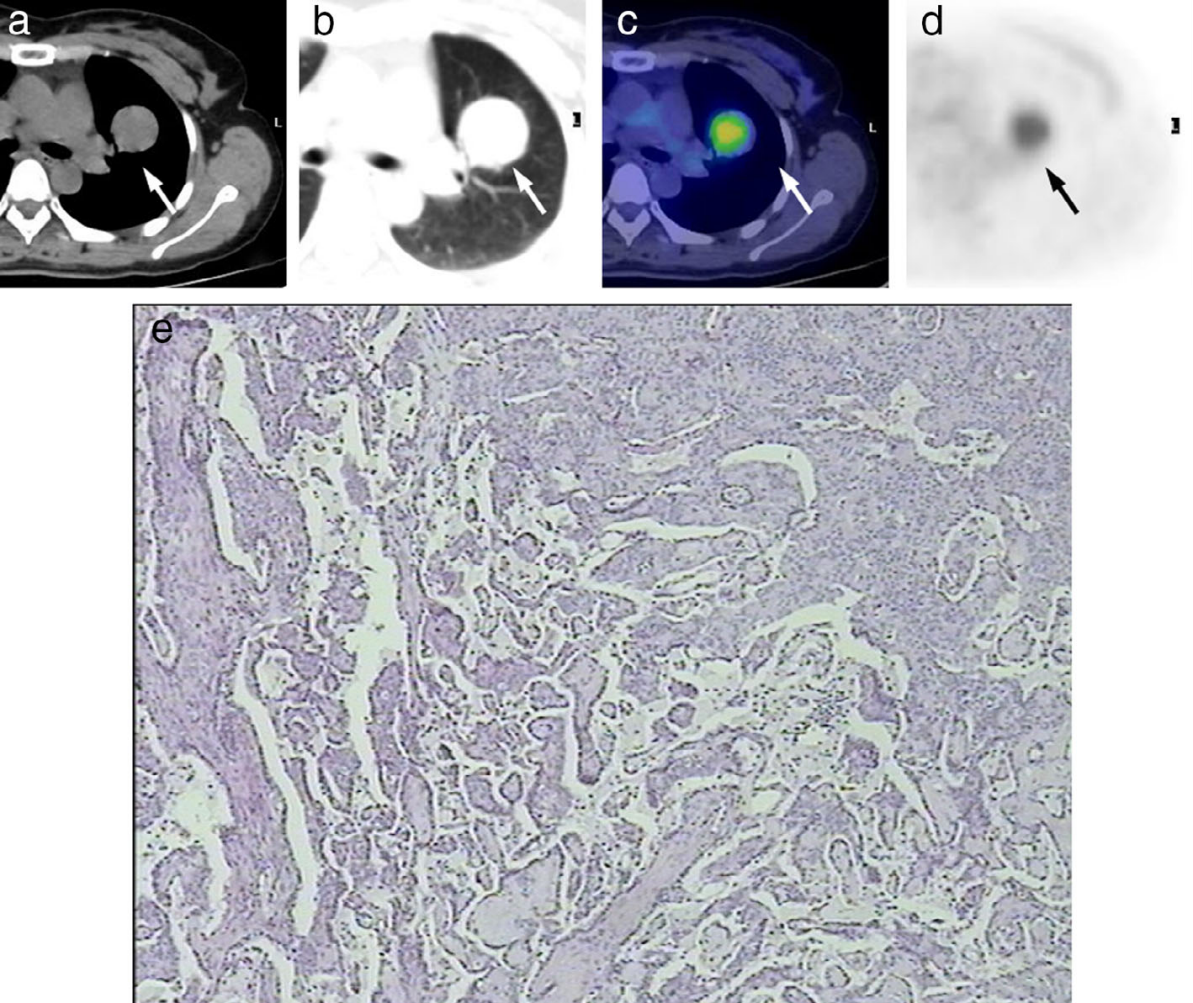
FDG PET/CT images and the corresponding histopathologic sections obtained in a 40‐year‐old woman (Case 1 in Table 1) with typical 3.3 cm PSPS in the left upper lobe (arrow). (**a**, **b**) CT examination showed typical PSPS manifestations, of a round solitary pulmonary mass with well‐circumscribed borders. (**c**, **d**) The corresponding PET and fused PET/CT images showed a relatively high uptake of FDG (SUV_max_ = 3.7). (**e**) Pathological images show pulmonary sclerosing hemangioma with active growth of alveolar epithelium (hematoxylin‐eosin stain; original magnification, x200).

**Figure 2:**
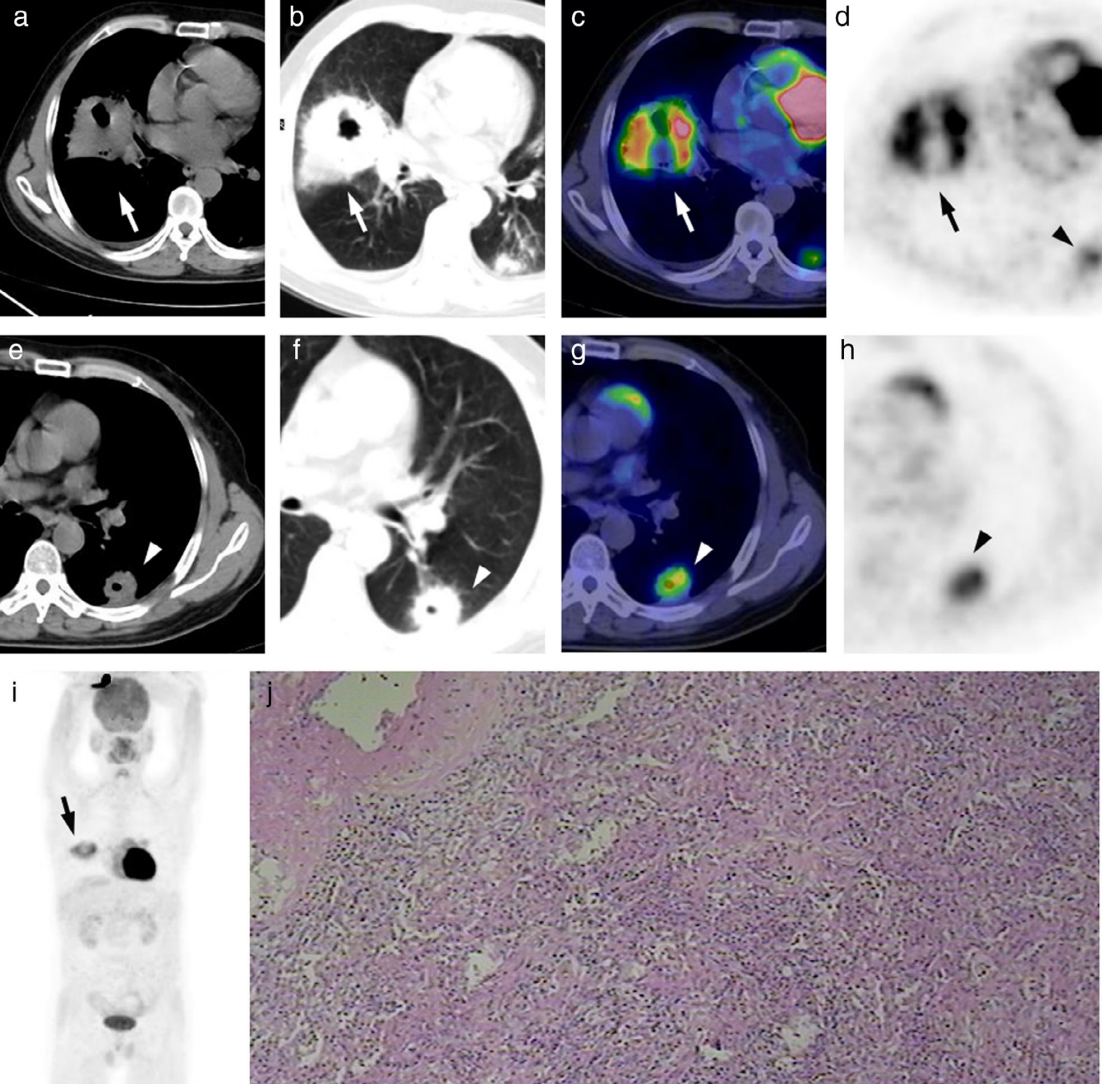
A 51‐year‐old man presented with a cough productive of yellow purulent sputum (Case 5 in Table 1). FDG PET/CT images showed two cavitary masses in the right middle lobe (**a**–**c**) (white arrow) and left lower lobe (**e**–**g**) (white arrowhead). The two lesions, showing intense uptake of FDG (SUV_max_ of 8.3, 4.9, respectively), can be seen in the same axial PET image (**d**) and (**h**) (black arrow/arrowhead). The right middle lobe lesion can be seen in the maximum intensity projection (MIP) image (**i**) (black arrow) while the left lower lobe lesion is obscured behind the heart. The right middle lobe lesion was pathologically confirmed as PSPs (**j**) (hematoxylin‐eosin stain; original magnification, x200), and the left lower lobe lesion had resolved on a two month follow‐up CT examination after anti‐inflammatory treatment.

**Figure 3:**
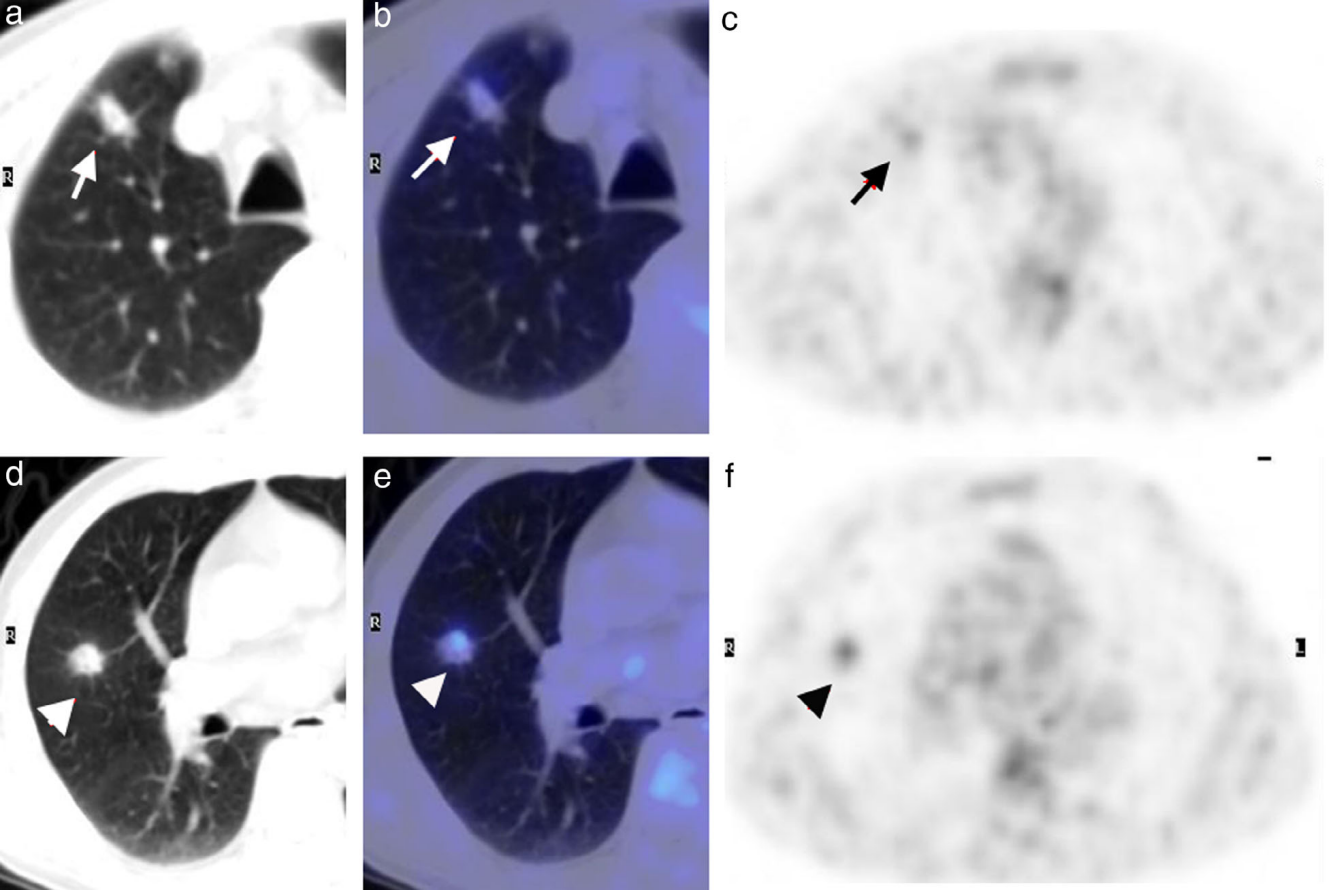
Two atypical PSPs were found in the right upper lobe (**a, b**) and right middle lobe (**d, e**) in one patient (Case 3 in Table 1). The two lesions showed moderate and intense uptake of FDG, respectively (**c**, **f**).

**Figure 4:**
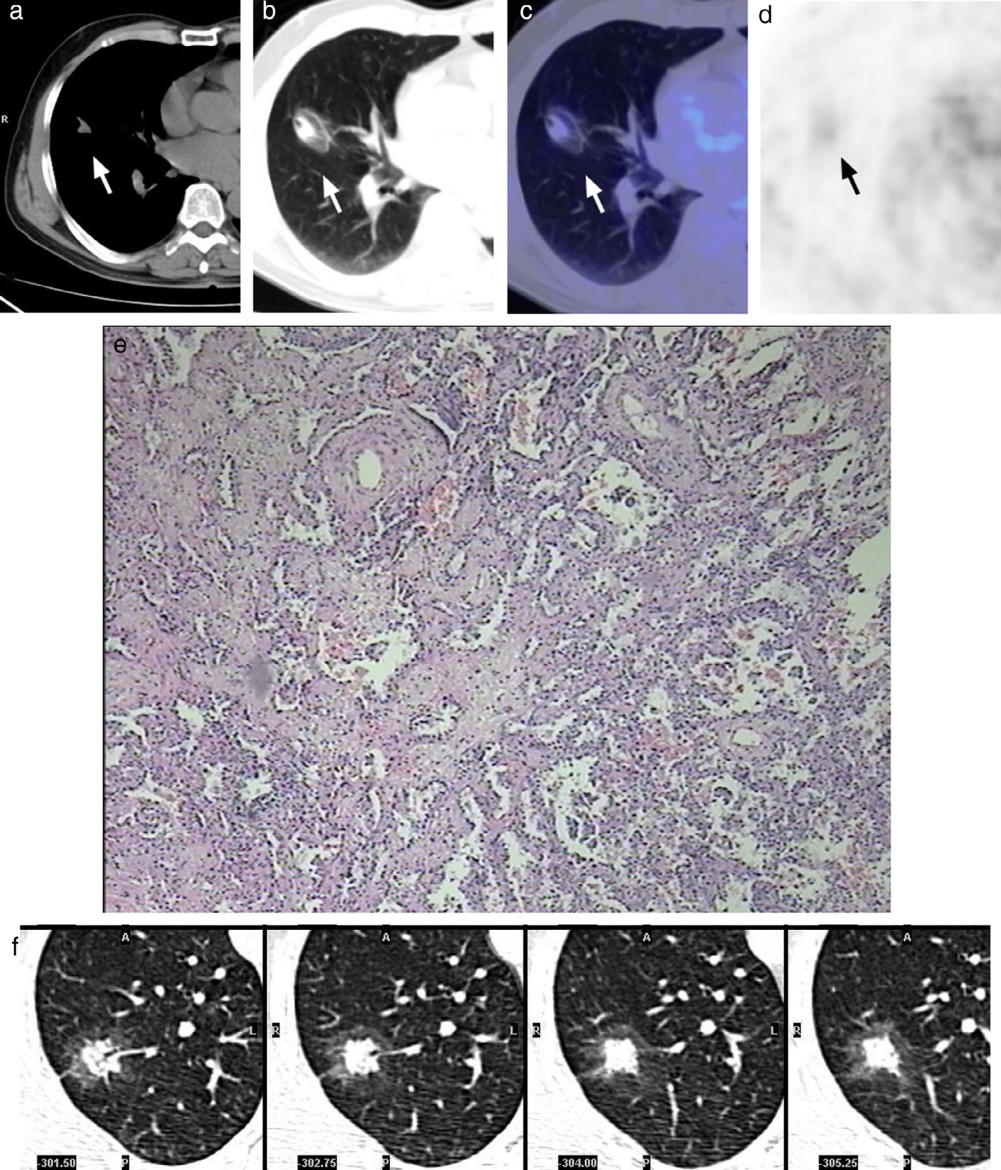
There was ground glass opacity (GGO) surrounding two PSP lesions (the “halo sign”), on the axial CT image in the lung window setting. Pathologically, PSPs were accompanied by MIA (**a**–**e**) and AAH (**f**) in two patients (Cases 2 and 7 in Table 1) (hematoxylin‐eosin stain; original magnification, x200) and both lesions showed moderate FDG uptake (Table 1).

**Figure 5:**
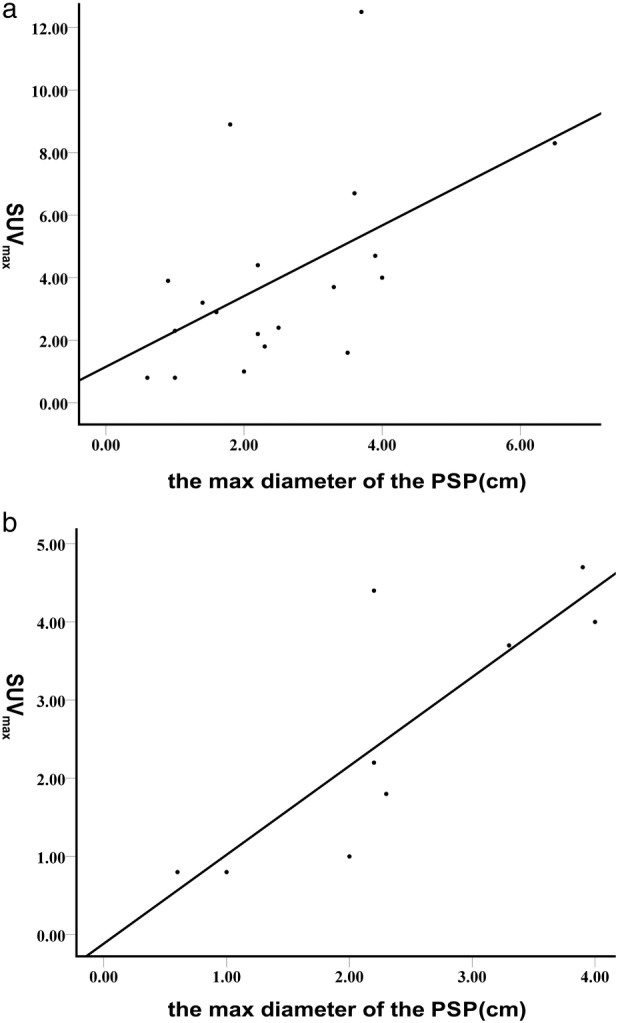
Linear regression analysis showed the correlation between the maximum diameter of PSP lesions and SUV_max_: (**a**) Correlation between the diameter of PSPs and SUV_max_ among all 23 PSPs, R = 0.518, R^2^ = 0.268, *P* = 0.011; (**b**) correlation between the diameter of PSPs and SUV_max_ in typical PSPs, R = 0.806, R^2^ = 0.650, *P* = 0.001).

PSPs that were round or oval in shape with well‐defined borders were defined as typical (Fig 1), and those that were irregular defined as atypical, such as those with coarse margin, lobulation, spiculation, and inclusion of cavities on CT images (Figs 2, 3, 4). In Table 1, accordingly, we define typical PSPs as “1” and atypical PSPs as “2.” Positive correlation was found between the diameter of PSPs and SUV_max_ among all 23 PSPs (R = 0.518, R^2^ = 0.268, *P* = 0.011) (Fig 5a).

#### Typical PSPs

As defined above, we assessed 13 typical PSPs which are shown in Figure 1. Positive correlation was found between the diameter and SUV_max_ in typical PSPs (R = 0.806, R^2^ = 0.650, *P* = 0.001) (Fig 5b). Among the typical PSP patients, a 68‐year‐old woman with PSP underwent two PET/CT scans on 21 January 2014 and 15 March 2015. Another PSP patient aged 17 years underwent a dual‐time‐point FDG‐PET/CT scan in order to differentiate it from other pulmonary malignancies as reported.^9^, ^10^ Both of the SUV_max_ of the PSPs increased in their second PET/CT scans (Table 1).

#### Atypical PSPs

Unlike typical PSPs, no correlation was found between the diameter of atypical PSPs and SUV_max_(R = 0.479, R^2^ = 0.229, *P* = 0.162). The highest SUV_max_ of an atypical PSP was 12.5, which was accompanied by suppurative inflammation. One atypical PSP manifested as a thick‐walled cavity with intense uptake of FDG; it was difficult to distinguish it from an inflammatory cavitary lesion in the left lower lobe in the same patient (Fig 2). Two PSPs were found to have the spicule sign in the same patient (Fig 3, Case 3 in Table 1). As shown in Figure 4, there were two lesions with ground glass opacity (GGO) in two other patients.

#### Clinical findings

The locations of the PSPs are listed in Table 2. The group with symptoms showed a higher SUV_max_ than the asymptomatic group (5.68 ± 3.63 vs. 2.76 ± 1.18, respectively, *P* = 0.002).

**Table 2:** Clinical characteristics of pulmonary sclerosing pneumocytoma

Clinical findings	Detail	Number	%
Location (lobe, per lesion)	Right upper lobe	6	26.09
	Right middle lobe	5	21.73
	Right lower lobe	5	21.73
	Left upper lobe	3	13.04
	Left lower lobe	4	17.39
Symptomatic (per patient)		9	40.90
	Fever	4	
	Cough	7	
	Chest (back) pain	4	
	Hemoptysis	2	
Malignant differentiation (per lesion)	AAH	1	11.11
	MIA	1	

AAH, atypical adenomatous hyperplasia; MIA, microinvasive adenocarcinoma.

Six patients had underlying malignancies. Breast cancer, ampullary cancer, and papillary thyroid carcinoma occurred in one case, lung and colon cancer were found in two cases. The pathological types of the two lung cancers were invasive adenocarcinoma and bronchioloalveolar carcinoma (also now classified as adenocarcinoma^3^). The invasive adenocarcinoma showed intense FDG uptake while the latter showed no abnormal uptake (SUV_max_ 7.3 and 0.6, respectively). Both cancers were in the same lobe as the PSP and were resected along with it. All the other underlying malignancies were pathologically proven to be adenocarcinomas.

As Table 1 shows, 14 months after the first PET/CT scan, an increasing diameter (from 2.0 cm to 2.2 cm) and SUV_max_ (from 1.0 to 2.2) of the tumor in the 68‐year‐old woman (Case 13) was found in the second PET/CT scan, and PSP was considered after surgery in combination with immunohistochemistry. Pathological results reviewed that the boundary between tumor tissue and peripheral lung tissue was not clear, and the focal alveolar epithelium showed atypical hyperplasia.

A slightly increased SUV_max_ (from 4.0 to 4.8) was acquired of the tumor when the second chest PET/CT scan 75 minutes after the routine scan in the 17‐year‐old girl. The diagnosis of PSP was confirmed after surgery, and no malignant signs were seen on histopathological examination (Table 1, Case 8).

Although CT scan was suggestive of malignancy, excisions of the two lesions (as shown in Fig 2) which showed signs of spiculation were performed and the characteristic pathologic features PSP showed without any other evidence of malignancy.

Two atypical lesions with the “halo sign” (as shown in Fig 4) showed a tendency of malignant differentiation into MIA (microinvasive adenocarcinoma) (Fig 4a–e) and AAH (atypical adenomatous hyperplasia) (Fig 4f).

Two cases were accompanied by tuberculosis (TB) in the right upper lobe and mediastinum, respectively. The former patient presented with symptoms of fever, cough and hemoptysis. The TB lesions showed intense FDG uptake (SUV_max_ = 6.6); however, the metabolism of the PSP was not that high (Case 13, SUV_max_ 2.0) (moderate uptake). PET/CT scan showed intense uptake in a mediastinal lymph node (SUV_max_ = 11.7) in the latter case, and pathologically, mediastinal lymph node TB was considered.

Immunohistochemical staining was performed in six PSPs, and we found that some tumor cells were immunopositive for epithelial membrane antigen (EMA) (3/6), thyroid transcription factor‐1 (TTF‐1) (6/6), vimentin (2/6), CD68 (2/6) and cytoskeleton 7 (CK‐7) (4/6).

## Discussion

Pulmonary sclerosing pneumocytoma is a relatively rare benign tumor of the lung which occurs predominantly in middle‐aged Asian women. It is extremely difficult to obtain an accurate diagnosis for PSP in Caucasian persons because of its low incidence.^11^ Based on its higher incidence in females, estrogen receptors were studied immunohistochemically.^12^, ^13^ Overexpression for ERbeta was found in 91.9% of PSP patients, compared with 45.8% in non‐small cell lung cancers.^13^ In the samples collected from 136 patients, women also accounted for the absolute majority (83.1%). In addition to this, in our study, we found ten10 atypical PSPs in our 22 patients who underwent PET/CT scans, all of which were in men. However, whether there is a relationship between atypical PSP and male sex hormones has not been reported.

There is a high probability of PSP diagnosis in female patients aged 40–60 years old with the following morphological characteristics on CT images. (i) Single round or oval masses with smooth borders. (ii) Homogeneous on unenhanced CT scan and homogeneous/heterogeneous on contrast‐enhanced. (iii) Cystic‐like area and coarse spotty calcification which was consistent with cholesterol clefts.^14^ (iv) The marginal pseudocapsule sign, which is the compressed lung parenchyma adjacent to the tumor. (v) Overlapping vessels corresponding to the drainage vessels of adjacent tumors. (vi) The air gap sign defined as a gap or crescent region without lung markings around the lesion. (vii) The “halo sign” was shown in the lung window settings presented as a lesion with surrounding GGO.^15^ Previous studies have suggested that dynamic contrast‐enhanced CT scanning is an effective means for radiological diagnosis of PSPs.^16^ However, most of these imaging features coexist in lung adenocarcinoma.

In our study, we found that the SUV_max_ was positively correlated with tumor size in typical PSPs. Dual‐time‐point FDG‐PET/CT imaging in a patient revealed an increasing SUV_max_ in the delayed scans. This is consistent with results of previous studies.^5^, ^6^, ^7^ However, in the case of atypical PSPs, there seems to be no correlation between the diameter and the SUV_max_. The highest SUV_max_ we found was 12.5, which was higher than any previously reported. There was no uptake of ^18^F‐FDG in three PSPs, the diameter of which were 1 cm, 0.6 cm and 2 cm, respectively. The SUV_max_ of the two smaller PSPs may be underestimated due to partial volume effect.^17^

A total of 13 tumors were found incidentally on chest radiographs, but the other nine patients presented with symptoms such as fever, chest pain, cough, and hemoptysis. The patients with symptoms showed a higher mean uptake of FDG than the asymptomatic group. Pathologically, the PSPs with the highest SUV_max_ were accompanied by suppurative inflammation. We assumed that the accompanying inflammation may be the main cause of a high uptake of FDG in PSPs. A misdiagnosis can be easily made without considering the symptoms. In our study, we also found that accompanying TB maybe another reason for the uptake.

Underlying malignancy, including breast cancer, ampullary cancer, papillary thyroid carcinoma, and colon cancer existed in five patients, which increased the difficulty of distinguishing between metastases from PSPs and second primary tumors.

The “halo sign,” most likely due to surrounding hemorrhage, is a typical CT sign in PSP.^15^ Another study of an atypical PSP found that tumor infiltration along the surrounding lung parenchyma may correspond with the GGO seen in the CT scans.^18^ This study showed progressive growth of PSP‐like TB. In our study, there were two atypical PSPs with the “halo sign”, pathologically proven to be PSP combined with AAH and MIA, respectively; however, imaging and pathological comparison study were not carried out due to the limitations of a retrospective study.

Recent studies reported a PSP with wax‐and‐wane pattern of growth, and it was suggested that the bleeding and resorption of PSP may have caused the change.^19^ In our study, a woman aged 68 showed increasing diameter and SUV_max_ of the tumor over 14 months. Pathologically, studies showed PSP combined with atypical hyperplasia in this tumor, implying a growth tendency of PSP.

Positive EMA, TTF‐1 and CK‐7 staining indicated that PSP originated from lung epithelial cells.^20^ Different levels of epithelial markers were also observed in our study.

Although in our hospital there was a considerable number of 136 patients with proven PSP, the limitation of our study was the small number of patients undergoing PET/CT examination.

In summary, the SUV_max_ of FDG uptake was positively correlated with the tumor size in typical PSP cases. More atypical PSPs were found in men than women and there was no correlation between SUV_max_ and the size of atypical PSPs. False‐positive results caused by atypical PSPs should be considered in lung cancer diagnosis through PET/CT imaging, especially those that are symptomatic.

## Disclosure

No authors report any conflict of interest.
